# Influence of Neighborhood Environment on Korean Adult Obesity Using a Bayesian Spatial Multilevel Model

**DOI:** 10.3390/ijerph16203991

**Published:** 2019-10-18

**Authors:** Eun Young Lee, Sugie Lee, Bo Youl Choi, Jungsoon Choi

**Affiliations:** 1 Institute for Health and Society, Hanyang University, Seoul 04763, Korea; 2 Department of Preventive Medicine, College of Medicine, Hanyang University, Seoul 04763, Korea; 3 Department of Urban Planning and Engineering, Hanyang University, Seoul 04763, Korea; 4 Department of Mathematics, Hanyang University, Seoul 04763, Korea

**Keywords:** neighborhood, environment, obesity, Bayesian spatial multilevel model, Korea

## Abstract

Previous studies using spatial statistical modeling that account for spatial associations between geographic areas are scarce. Therefore, this study examines the association between neighborhood environment and obesity using a Bayesian spatial multilevel model. Data from 78,014 adults living in Gyeonggi province in Korea were drawn from the 2013–2014 Korean Community Health Survey. Korean government databases and ArcGIS software (version 10.1, ESRI, Redlands, CA) were used to measure the neighborhood environment for 546 administrative districts of Gyeonggi province. A Bayesian spatial multilevel model was implemented across gender and age groups. The findings indicate that women aged 19–39 years who lived in neighborhoods farthest away from parks were more likely to be obese. Men aged 40–59 years who lived in neighborhoods farther from public physical activity facilities and with lower population density were more likely to be obese. Obesity for women aged 19–39 years was the most spatially dependent, while obesity for women aged 40–59 years was the least spatially dependent. The results suggest that neighborhood environments that provide more opportunities for physical activity are negatively related to obesity. Therefore, the creation of physical activity in favorable neighborhood environments, considering gender and age, may be a valuable strategy to reduce obesity.

## 1. Introduction

The prevalence of obesity has increased globally. Between 1975 and 2014, the mean body mass index (BMI) has increased by 0.59–0.63 kg/m^2^ per decade in women and men [1]. Additionally, the age-standardized prevalence of obesity has increased by 7.6% in men and 8.5% in women from 1975 to 2014 [1]. Being obese is one of the greatest risk factors for type 2 diabetes, cardiovascular disease, certain cancers, and premature death [2]. In 2010, high BMI was estimated to cause 3.4 million deaths and 3.8% of global disability-adjusted life years (DALYs) [3]. Similarly, the prevalence of obesity among Korean adults has increased from 25.8% in 1998 to 32.8% in 2012 [4]. Moreover, the total socioeconomic costs of being overweight and obese in Korea in 2015 were approximately 0.22% of gross domestic product (GDP) and 3.7% of national health care expenditure [5].

In addition to individual-level factors such as genetic predisposition, sociodemographic factors, and lifestyle [2,6,7], neighborhood environment has been identified as a key factor in explaining the current global epidemic of obesity [8,9,10]. Fundamentally, obesity is caused by an imbalance between energy intake and expenditure. Physical activity (PA) increases energy expenditure to prevent or reduce unwanted weight gain and may also attenuate genetic susceptibility to obesity [11]. Most studies have reported that a high level of PA and a low level of BMI are associated with activity-supporting neighborhood characteristics such as walkability; the important characteristics of such environments include residential density, land use mix, and street connectivity [8,12,13]. Residential density has relevance in supporting policies for compact, mixed-use urban development and discouraging automobile-oriented urban design [14]. Denser interconnected streets and a greater diversity of destinations within walking or biking distance may facilitate physical activity and reduce BMI. Moreover, convenient access to parks, recreational facilities, and transport infrastructure has also been associated with a high level of PA and a low level of BMI [12,15,16]. Proximity to parks and recreational facilities provides more opportunities for residents to engage in physical activity. Proximity to bus stops and subway stations encourages transportation-related walking and lower car use [17,18,19]. These findings indicate that activity-supporting neighborhood environments are more likely to reduce obesity.

Although there is a large body of literature examining the association between neighborhood environment and obesity, the existing research has several limitations. The influence of neighborhood environment on obesity differs according to gender and life stage among adults [20]. However, few studies have addressed this difference [20,21]. Moreover, because most studies have reported data from North America, Australia, and Europe [8,19], information on the association between neighborhood environment and obesity in Asian countries is insufficient. Lastly, most studies have used multilevel models to account for the nested structures of individuals within geographical areas [6]. However, neighborhood physical environment features are not independent of geographic area, and health is influenced by the surrounding neighborhood. Although multilevel models allow for the simultaneous estimation of individual and area-level effects, they cannot account for spatial associations between geographic areas [9,22]. In contrast, the Bayesian spatial multilevel model, which was developed to incorporate spatial association, is an appropriate method to examine the influence of neighborhood spatial variables on obesity [22]. To our knowledge, however, no previous studies have investigated the relationship between neighborhood environment and obesity using the Bayesian spatial multilevel model. Therefore, the goal of the present study is to examine the influence of neighborhood physical environment on obesity according to gender and age among adults in the Gyeonggi province of Korea using the Bayesian spatial multilevel model to account for spatial association.

## 2. Materials and Methods

### 2.1. Participants and Study Area

Individual data were collected from the 2013–2014 Korean Community Health Survey (KCHS) in Gyeonggi province, which surrounds Seoul, the capital of South Korea. Gyeonggi province includes 546 administrative districts with an average population of 28,747 in 2013 and has a mixture of urban, suburban, and rural areas. This province consists of approximately 24% (12.98 million in 2016) of the total population of South Korea. The KCHS is a community-based cross-sectional survey used to provide community health statistics for adults aged 19 years or older. Approximately 230,000 individuals participate in the KCHS each year, using multistage probability sampling from the registered population data of the Ministry of Public Administration and Security throughout the country [23]. After obtaining written informed consent for the survey, data were collected through a computer-assisted personal interview by trained interviewers using a standardized questionnaire [23]. Detailed information on the KCHS has been previously described [23]. The Institutional Review Board of the Korea Centers for Disease Control and Prevention approved the protocols of the KCHS (2013-06EXP-01-3C and 2014-08EXP-09-4C-A). A total of 82,419 individuals living in Gyeonggi province participated in the 2013 and 2014 KCHS. After excluding data with insufficient information, a total of 78,014 individuals from the 546 administrative districts of Gyeonggi province were used in the present study.

### 2.2. Measures

#### 2.2.1. Obesity

Body mass index (BMI = weight (kg)/height (m)^2^) was calculated using self-reported height and weight in the survey and categorized into two groups: nonobese (<25.0 kg/m^2^) and obese (≥25.0 kg/m^2^) [24].

#### 2.2.2. Neighborhood Environment

Distances to public PA facilities, parks, and transit; densities of population and intersections; and land use mix (LUM) were measured by using a geographic information system (GIS) to characterize the neighborhood environment. Korean government databases and ArcGIS software (version 10.1, ESRI, Redlands, CA) were used to measure the neighborhood environment for the 546 administrative districts of Gyeonggi province, which was the smallest spatial unit. Four sources of information were used in the present study: The National Public PA Facility Database 2013 of the Ministry of Culture, Sports, and Tourism; the Korean Transport Database 2013 of the Ministry of Land, Infrastructure and Transport; the National Building Database 2013 of the Ministry of Land, Infrastructure, and Transport; and the Population Census 2013 of Statistics Korea. All databases provide annual data, except for the National Building Database, which provides monthly data.

We first classified the urbanized land area from each of the 546 administrative spatial units as developed land areas excluding mountain areas, watershed areas, and natural open spaces. The urbanized land area of each administrative spatial unit was divided into grid cells of 100 by 100 m. Then, the average distance (i.e., the Euclidean distance) between the centers of the 100 × 100 m grids of the urbanized land area and the nearest destinations, such as public PA facilities (e.g., athletic field, football field, tennis court, or swimming pool), public parks, or public transit (i.e., a bus stop or a subway station) were calculated for each administrative spatial unit. The population density for each administrative spatial unit was calculated as the population divided by the urbanized land area.

The intersection density was expressed as the number of intersections with three or more legs divided by the urbanized land area. Lastly, LUM was calculated using the entropy index, which is the ratio of domestic, commercial, and business building floor area divided by the urbanized land area [25]. The values of LUM ranged from 0 (lowest heterogeneity of land use) to 1 (highest heterogeneity of land use). Because of the nonlinear effects of neighborhood environment on obesity, all neighborhood environment variables were categorized into three tertiles, ranging from the lowest number in tertile 1 to the highest number in tertile 3.

#### 2.2.3. Individual Factors

Socio-demographic factors included gender, age (19–39 years, 40–59 years, or ≥60 year), education (≤high school or ≥college), monthly household income (<2793 USD or ≥2793 USD, exchange rate 1 USD = 1074.2 won), job (nonmanual, manual, or other including housewife, soldier, college student, or unemployed), and marital status (live with/without spouse).

Health behavior factors included smoking and drinking behaviors (never, former, or current), sleeping duration (<7 h/d or ≥7 h/d), moderate and vigorous physical activity (MVPA) (no/yes), level of sodium in the diet (high, middle, or low) and driving a car (no/yes). The International Physical Activity Questionnaire–Short Form (IPAQ-SF) [26] was used to obtain self-reported PA over the previous 7 days. Participants who performed vigorous-intensity activities for at least 20 min per day on 3 or more days, moderate-intensity activities and/or walking for at least 30 min per day on 5 or more days, or any combination of walking, moderate-intensity or vigorous-intensity activities achieving a minimum of at least 600 metabolic equivalent for task (MET)-min per week on 5 or more days were categorized into the MVPA group. Participants who responded “very salty” or “a little salty” to the item “How do you usually eat?” were classified as participants who eat a high level of sodium in the diet [27]. The participants who responded “bland” or “very bland” to the same item were classified as participants who eat a low level of sodium in the diet [27].

Health status factors included subjective health status (poor/good), stress perception (no/yes), depressive symptoms (no/yes), and number of chronic illnesses (e.g., hypertension, diabetes, dyslipidemia, stroke, myocardial infarction, angina, and arthritis) (0, 1, or ≥2). Participants who reported a moderate or high degree of stress were classified into the stress group. Depression was determined by a ‘‘yes’’ response to the item ‘‘During the last a year, were you feeling so sad or hopeless for at least 2 weeks or more that you stopped doing some usual activities?’’

### 2.3. Statistical Analyses

We first investigated the effects of the individual-level factors of the study participants and the neighborhood environmental factors on obesity. Since factors related to obesity vary with age group and gender, the analyses were conducted separately by age group (i.e., 19–39 years old, 40–59 years old, 60 years old and older) and gender. To identify the obesity risk factors for each age and gender group, the bivariate relationships between the individual and neighborhood environmental factors and obesity were assessed by using univariate logistic regression models.

A Bayesian multilevel model with spatial random effects was implemented because it provided a number of advantages. Multilevel modeling incorporates data at multiple levels of analysis simultaneously to estimate level-specific effects. In this study, we investigated the effects of individual-level factors as well as area-level (neighborhood environment) factors on obesity. In addition, the Bayesian multilevel model can account for complex dependency structures in multilevel data, such as geographical variations of the log-odds of obesity.

We let $y_{ij}$ be the binary obesity variable for individual $j\;\left(=1,\ldots ,n_{i}\right)$ of neighborhood i (=1,…,N) following a Bernoulli distribution with the probability of obesity, $p_{ij}$

(1) $$y_{ij}\;\sim \;Bernoulli\;\left(p_{ij}\right)$$

(2) $$logit\;\left(p_{ij}\right)\;=\;\log\;\left(\frac{p_{ij}}{1-p_{ij}}\right)=\lambda_{ij}.$$

For each model, $\lambda_{ij}$ is specified as follows:

Model 1: $\lambda_{ij}=\;\alpha \;+v_{i}$
Model 2: $\lambda_{ij}=\;\alpha \;+\;X_{ij}^{\prime }\beta \;+\;v_{i}$
Model 3: $\lambda_{ij}=\;\alpha \;+\;X_{ij}^{\prime }\beta \;+\;W_{i}^{\prime }\gamma \;+\;v_{i}$
Model 4: $\lambda_{ij}=\;\alpha \;+\;X_{ij}^{\prime }\beta \;+\;W_{i}^{\prime }\gamma \;+\;u_{i\;}+\;v_{i}$

Here, α is the intercept parameter, and $v_{i}$ is the spatially uncorrelated random component to capture the spatially independent variation following an independent normal distribution with the mean 0 and variance $\sigma_{v}^{2}$, N (0, $\sigma_{v}^{2}$). The covariates $X_{ij}^{\prime }$ and $W_{i}^{\prime }$ are the individual factors and the neighborhood environmental factors, respectively. The corresponding coefficients β and γ are the vectors of the individual-factor effects and the neighborhood environmental-factor effects, respectively. The spatially correlated random component $u_{i}$ follows the conditional autoregressive distribution [28] expressed as follows:

(3) $$u_{i}|u_{k},\;k\;\neq \;i\;\sim \;N\;\left(\underset{k}{\sum }\frac{w_{ik}u_{k}}{w_{i+}},\;\frac{\sigma_{u}^{2}}{w_{i+}}\right),\;i,k\;=\;1,2,\cdots ,N.$$

The neighborhood information $w_{ik}$ is 1 if the ith region and kth region are adjacent, and 0 otherwise. The number of regions adjacent to the ith region is denoted by $w_{i+}$. For computational efficiency, integrated nested Laplace approximations (INLAs) and Bayesian estimation techniques were implemented using the R package (R-INLA) [29] when fitting these models. The intercept and fixed parameters were assigned a vague prior, N (0, 10,000). Prior distributions for $\sigma_{v}^{2}$ and $\sigma_{u}^{2}$ were specified as

(4) $$\log\left(\frac{1}{\sigma_{v}^{2}}\right),\;\log\left(\frac{1}{\sigma_{u}^{2}}\right)\;\sim \;logGamma\;\left(1,\;0.0005\right),$$

which are vague priors used for precision parameters. These are the default prior distributions in R-INLA [29].

Model 4 was selected as the best model among the candidate models using the deviance information criterion (DIC) [30], which is commonly used in Bayesian selection problems like the Akaike information criterion (AIC). The DIC is defined by

(5) $$DIC=pD+\bar{D\left(\theta \right)},$$

where θ denotes the vector of the parameters, $\bar{D\left(\theta \right)}$ is the posterior mean of deviance, D(θ)=−2log(y|θ), and pD is the complexity of the model obtained from $pD=\bar{D\left(\theta \right)}-D\left(\hat{\theta }\right)$, where $\hat{\theta }$ is the posterior mean of θ. A model with a smaller DIC value has better goodness of fit. In addition, the spatial fraction (SF) was calculated to assess the extent to which the unexplained variation is associated with the geographical location. The SF is defined by

(6) $$SF=\frac{\sigma_{u}^{2}}{\sigma_{u}^{2}+\sigma_{v}^{2}}.$$

An SF value close to one would indicate that a spatial-dependent effect dominates [22].

To investigate the effects of the prior selection in the results, we conducted a sensitivity analysis with prior distributions of $\sigma_{v}^{2}$ and $\sigma_{u}^{2}$ as

(7) $$\log\left(\frac{1}{\sigma_{v}^{2}}\right),\;\log\left(\frac{1}{\sigma_{u}^{2}}\right)\sim logGamma\left(1,\;1\right).$$

A model with these prior distributions also provided almost similar results.

## 3. Results

**Figure 1:**
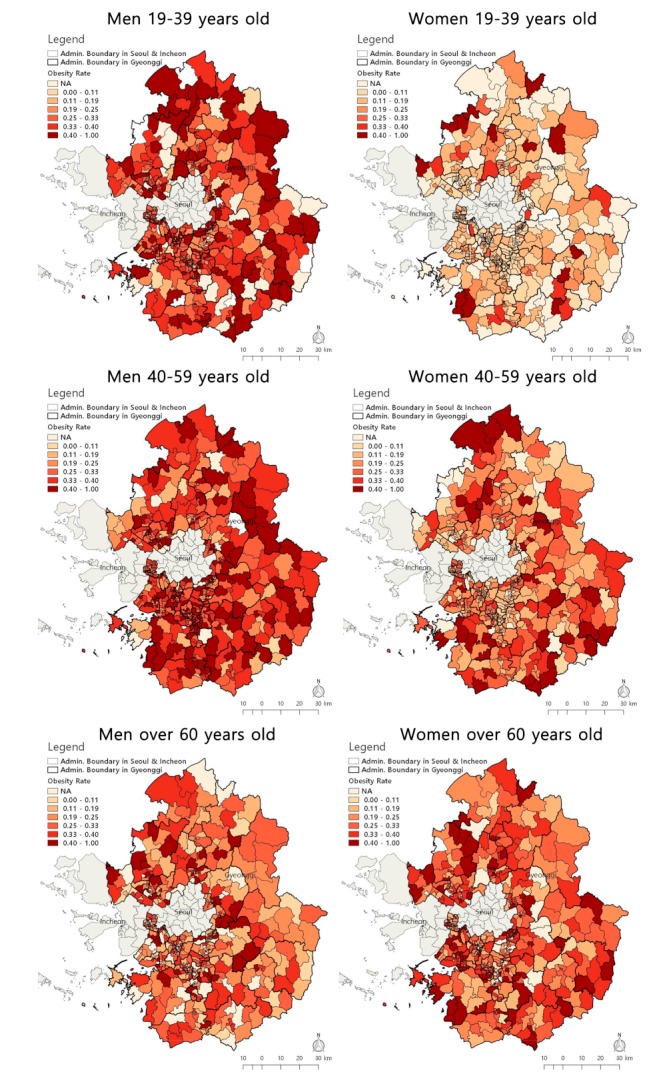
Spatial distribution of obesity rate by gender and age.

The general characteristics of the study participants and neighborhood physical environments for obesity are shown in Table 1. Approximately 25.4% of the participants were obese. Of the 19,782 obese participants, 11,585 (58.6%) were males. For the obese participants, 9162 (46.4%) and 5823 (29.4%) were 40–59 years old and 19–39 years old, respectively. More obese participants lived in neighborhoods with longer distances to public PA facilities, public parks, and public transit; lower population densities and intersection densities; and a higher LUM compared to nonobese participants. Figure 1 shows the spatial variations of the obesity rates by gender and age.

Through univariate logistic regression models, we found that different gender and age groups had different relationships between individual and neighborhood environmental factors and obesity (data not presented). Among the neighborhood environmental factors, distance to public PA facilities and LUM were related to obese men 19–39 years old. The distance to public PA facilities and population density were related to obese men 40–59 years old, and only distance to public transit was related to obese men over 60 years old. All neighborhood environmental factors for women 40–59 years old were related to obesity, while no neighborhood factors were related to obesity for women over 60 years old. For women 19–39 years old, all neighborhood environmental factors except intersection density were related to obesity. From the univariate analysis, most of the individual factors considered in this study were related to obesity, but the relationship between individual factors and obesity was different for different age and gender groups. Significant variables were included in the next step of the analysis. 

The goodness of fit indexes of the four models were calculated by gender and age group (data not presented). For men over 60 years old, the DIC value decreased from 8914.45 for Model 1 to 8613.18 for Model 4. In the other two groups of men, Model 4 had the smallest DIC value. Furthermore, after adjusting for neighborhood environmental factors and spatial variation in women 40–59 years old, the DIC value decreased from 18,103.00 to 17,077.72. For women 19–39 years old and over 60 years old, Model 4 had the smallest DIC value. This result indicates that neighborhood environmental factors are related to obesity in addition to the extra spatial structure. Table 2 presents the association between obesity and the individual and neighborhood environmental factors from the Bayesian spatial multilevel model (Model 4) by gender and age group. After considering the individual and neighborhood environmental factors as well as the spatial variation in the model, living in neighborhoods with a middle-level distance to public PA facilities (Tertile 2) was associated with a 13% increase in odds for obesity in men 40–59 years old, compared with men living in neighborhoods nearest to public PA facilities (OR = 1.13, 95% CI = 1.03–1.23 for Tertile 2 vs. Tertile 1). In addition, obesity rates were lower among men 40–59 years old living in neighborhoods with a middle-level population density (Tertile 2) compared with those living in neighborhoods with the lowest population density (OR = 0.88, 95% CI = 0.79–0.98 for Tertile 2 vs. Tertile 1). Women 19–39 years who lived in neighborhoods farthest from public parks significantly influenced obesity (OR = 1.33, 95% CI = 1.05–1.69 for Tertile 3 vs. Tertile 1). For women 40–59 years, no neighborhood physical environmental factors significantly influenced obesity. In addition, the SF value indicates the proportion of variability due to the spatial dependence in the random effects. For female groups 19–39 years old, the value was over 99%, which means that no strong spatial dependent structure was captured by the risk factors, while the SF value for females 40–59 years old was only 3.8%. For the other groups, the SF values were about 46.7% to 56.5%, indicating that there was a fair amount of spatial dependence and spatial heterogeneity. Among the individual factors, job, smoking, driving a car, and number of chronic illnesses were significantly associated with obesity in men in all age groups, while diet and number of chronic illnesses were significantly associated with obesity in all women.

## 4. Discussion

Using a Bayesian spatial multilevel model, the influence of neighborhood environment on obesity was assessed by gender and age with a large sample from the KCHS. This study revealed that neighborhood environments that provided more opportunities for PA were negatively related to obesity. Women aged 19–39 years who lived in neighborhoods farthest from public parks were more likely to be obese. The findings are in line with previous studies [15,31] that have reported more benefits of parks on weight status for women than men. For men aged 40–59 years, men who lived in neighborhoods further from public PA facilities were more likely to be obese, while men who lived in neighborhoods with higher population density were less likely to be obese. These findings are somewhat inconsistent with a recent study using data from the UK Biobank [32] that found larger estimates of the effects of PA facilities on women’s BMI than on men’s BMI. Additionally, a study in the US [33] reported a stronger association among women than men between obesity and high population density, while Frank and colleagues [18] reported a stronger association among white men than women. Because of the lack of evidence for an association between neighborhood environments and weight status by gender and age [8], the findings warrant further study to fully understand the complex relations involved.

The SF values for men were less than 50% and were similar in all age groups. However, the values for women differed between age groups. For women aged 19–39, 99% of the variance in the fully adjusted Bayesian spatial model was spatially structured, while only 3% of the variance was spatially structured for women aged 40–59. Thus, obesity for women aged 19–39 years was the most spatially dependent, while obesity for women aged 40–59 years was the least spatially dependent. There are some possible explanations for this difference. First, younger women are under more social pressure to lose weight and stay thin [34] and are more responsive to the opinions and behaviors of others [35]. Thus, younger women are more affected by their community than men and older women. Second, middle-aged women are less likely to participate in the labor market than younger women as well as men and are more likely to focus on parenting. In fact, only half of Korean women over 15 years old are employed, and women over 40 years old are mostly part-time workers [36]. Thus, middle-aged women tend to spend more time in their localities and are more like to be exposed to the neighborhood environment [37].

Regarding individual factors, men with high socioeconomic status (i.e., a college degree, nonmanual labor, and car user) were more likely to be obese, while women with high socioeconomic status were less likely to be obese. These findings are consistent with previous studies [7,38]. This study added that these associations were more pronounced for the 40–59 age group than the other groups. Furthermore, both men and women in the 40–59 age group who slept more than seven hours, were involved in MVPA, and had a low sodium diet were less likely to be obese. A possible explanation for our results is that the middle-aged group participates more in healthy behavior because they recognize the social and biological process of aging (e.g., menopause). On the other hand, men and women aged 19–39 years who were living with a spouse, former smokers, and had poor subjective health status and high stress perception were more likely to be obese. An inverse association between smoking and obesity was found in the male group, while an association between alcohol consumption and obesity was reported in the female group. Lastly, this study revealed a positive association between chronic disease and obesity across gender and age group. This finding is consistent with a previous study [38].

To our knowledge, the present study is the first to assess the influence of neighborhood physical environment on obesity using a Bayesian spatial model to account for the spatial association. Additionally, this study revealed the association between objectively measured neighborhood environments and obesity according to gender and age using a large sample from the KCHS. Despite the strengths of current study, there are limitations. First, this is a cross-sectional design where no causality should be assumed from the results. Second, height and weight were measured by self-report data, although the data were obtained through a face-to-face interview by trained interviewers. Since people tend to underreport their weight and overreport their height [39], the possibility of underestimating BMI in this study cannot be ruled out. Third, although BMI is a common measure of obesity in population-based studies, it does not distinguish between lean body mass and body fat mass [40]. Future research should consider including central obesity measures (i.e., waist circumference, waist:hip ratio and waist:height ratio) because these are associated with morbidity and mortality independently of BMI. Lastly, other neighborhood characteristics supporting healthy eating and the surrounding workplace were not investigated.

## 5. Conclusions

The present study highlights the importance of PA-favorable neighborhood environments (i.e., proximity of PA facilities and parks and high population density) to reduce the sharp increase in obesity in Korean adults. The association between neighborhood environment, including spatial variation, and obesity varied according to gender and age. The findings suggest that creating more PA-favorable neighborhood environments that consider the gender and age of the residents may be a valuable strategy to reduce obesity. Although the current study focuses on spatial variation as well as neighborhood environmental factors for the 2013–2014 data drawn from KCHS, future studies are needed to further investigate the spatio-temporal association between obesity and individual- and neighborhood-level factors by using a Bayesian space–time multilevel model.

## Figures and Tables

**Table 1:** Characteristics of the study participants and the neighborhood environment.

Variables	Nonobese (N = 58,232)	Obese (N = 19,782)
	n (%)	n (%)
**Sociodemographic characteristics**		
Gender		
Male	24,536 (42.1)	11,585 (58.6)
Female	33,696 (57.9)	8197 (41.4)
Age		
19–39 years old	21,251 (36.5)	5823 (29.4)
40–59 years old	24,285 (41.7)	9162 (46.4)
60 years old and older	12,696 (21.8)	4797 (24.2)
Education		
≤High school	30,843 (53.0)	11,685 (59.1)
≥College	27,389 (47.0)	8097 (40.9)
Household income		
<2793 USD	23,765 (40.8)	8460 (43.7)
≥2793 USD	34,467 (59.2)	11,142 (56.3)
Job		
Nonmanual job	15,749 (27.1)	5379 (27.2)
Manual job	19,466 (33.4)	7794 (39.4)
Other	23,017 (39.5)	6609 (33.4)
Marital status		
Live with spouse	45,959 (78.9)	16,158 (81.7)
Live without spouse	12,273 (21.1)	3624 (18.3)
**Health behaviors**		
Smoking		
Never smokers	38,108 (65.4)	10,510 (53.2)
Former smokers	8158 (14.1)	4082 (20.6)
Current smokers	11,966 (20.5)	5190 (26.2)
Drinking		
Never drinkers	8200 (14.1)	2710 (13.7)
Former drinkers	6828 (11.7)	2303 (11.6)
Current drinkers	43,204 (74.2)	14,769 (74.7)
Sleeping duration		
<7 h/d	27,302 (46.9)	10,292 (52.0)
≥7 h/d	30,930 (53.1)	9490 (48.0)
Moderate and vigorous physical activity		
No	20,361 (35.0)	7249 (36.6)
Yes	37,871 (65.0)	12,533 (63.4)
Diet		
High-sodium diet	14,463 (24.8)	6094 (30.8)
Middle-sodium diet	28,438 (48.8)	9387 (47.5)
Low-sodium diet	15,331 (26.4)	4301 (21.7)
Driving a car		
Yes	31,921 (54.8)	12,248 (61.9)
No	26,311 (45.2)	7534 (38.1)
**Health status**		
Subjective health status		
Poor	7908 (13.6)	3534 (17.9)
Good	50,324 (86.4)	16,248 (82.1)
Stress perception		
No	42,009 (72.1)	13,443 (68.0)
Yes	16,223 (27.9)	6339 (32.0)
Depressive symptoms		
No	54,312 (93.3)	18,408 (93.1)
Yes	3920 (6.7)	1374 (6.9)
Number of chronic illnesses		
0	42,308 (72.7)	10,930 (55.2)
1	9584 (16.5)	4489 (22.7)
≥2	6340 (10.8)	4363 (22.1)
**Neighborhood environment (median; min–max)**		
Distance to public physical activity facilities (m)		
T1 (511.9; 205.3–742.2)	20,451 (35.1)	6425 (32.5)
T2 (1020.6; 742.2–1525.6)	22,396 (38.5)	7742 (39.1)
T3 (2406.7; 1525.6–9783.6)	15,385 (26.4)	5615 (28.4)
Distance to public parks (m)		
T1 (119.7; 39.9–169.5)	21,117 (36.3)	6683 (33.8)
T2 (247.8; 169.5–469.6)	20,666 (35.5)	6957 (35.2)
T3 (1530.5; 469.6–6183.3)	16,449 (28.2)	6142 (31.0)
Distance to public transit (m)		
T1 (126.8; 85.1–158.9)	19,550 (33.6)	6220 (31.4)
T2 (219.9; 158.9–295.3)	22,519 (38.7)	7804 (39.5)
T3 (413.7; 295.3–1751.6)	16,163 (27.7)	5758 (29.1)
Population density (person per km^2^)		
T1 (15; 4.2–76.2)	15,100 (25.9)	5725 (28.9)
T2 (189; 76.2–285.7)	22,652 (38.9)	7613 (38.5)
T3 (395; 285.7–8067.8)	20,480 (35.2)	6444 (32.6)
Intersection density (intersection per km^2^)		
T1 (0.06; 0.00–0.09)	14,853 (25.5)	5281 (26.7)
T2 (0.15; 0.09–0.22)	21,861 (37.5)	7549 (38.2)
T3 (0.35; 0.22–2.89)	21,518 (37.0)	6952 (35.1)
Land use mix		
T1 (0.61; 0.02–0.70)	19,801 (34.0)	6326 (32.0)
T2 (0.77; 0.70–0.84)	19,613 (33.7)	6675 (33.7)
T3 (0.90; 0.84–1.00)	18,818 (32.3)	6781 (34.3)

n (%) numbers and percentages; USD, United States dollar; T, tertile.

**Table 2:** Association between obesity and selected individual and neighborhood environmental factors using a Bayesian multilevel logistic model across gender and age.

	Men						Women					
Variables	19–39		40–59		≥60		19–39		40–59		≥60	
	OR	95% CI	OR	95% CI	OR	95% CI	OR	95% CI	OR	95% CI	OR	95% CI
Education (ref. ≤ High school)												
≥College	0.95	(0.86,1.05)	**1.12 **	**(1.04,1.22)**			**0.60 **	**(0.53,0.68)**	**0.69 **	**(0.62,0.76)**	0.81	(0.66,1.00)
Household income (ref. < 3 million won)												
≥3 million won			1.07	(0.99,1.16)	1.09	(0.97,1.23)	**0.83 **	**(0.74,0.93)**	0.92	(0.84,1.00)	0.97	(0.87,1.07)
Job (ref. nonmanual labor)												
Manual labor	0.97	(0.88,1.06)	**0.90 **	**(0.83,0.98)**	0.90	(0.74,1.09)	**1.22 **	**(1.04,1.44)**	1.07	(0.95,1.21)	1.37	(0.87,2.21)
Other	**0.75**	**(0.66,0.84)**	**0.75 **	**(0.64,0.88)**	**0.73 **	**(0.61,0.89)**	**1.42 **	**(1.25,1.60)**	1.01	(0.90,1.14)	1.29	(0.83,2.07)
Marital status (ref. live with spouse)												
Live without spouse	**0.79 **	**(0.72,0.86)**			0.90	(0.75,1.08)	**0.69 **	**(0.60,0.80)**	0.91	(0.81,1.02)		
Smoking (ref. never smokers)												
Former smokers	**1.15**	**(1.02,1.30)**	**1.14 **	**(1.03,1.25)**	0.97	(0.86,1.10)	**1.65 **	**(1.32,2.04)**				
Current smokers	1.05	(0.96,1.15)	**0.83 **	**(0.76,0.91)**	**0.68 **	**(0.58,0.79)**	1.03	(0.81,1.31)				
Drinking (ref. never drinkers)												
Former drinkers	1.26	(0.96,1.66)					1.26	(0.99,1.60)	**1.16 **	**(1.02,1.33)**	1.08	(0.95,1.22)
Current drinkers	1.03	(0.85,1.26)					1.08	(0.88,1.33)	0.97	(0.88,1.08)	**1.25 **	**(1.13,1.39)**
Sleeping duration (ref. <7 h)												
≥7 h	**0.86**	**(0.80,0.94)**	**0.91**	**(0.85,0.98)**	0.91	(0.82,1.01)			**0.89 **	**(0.82,0.96)**		
MVPA (ref. no)												
Yes	0.97	(0.89,1.05)	**0.90 **	**(0.84,0.96)**	**0.86 **	**(0.77,0.96)**			**0.87 **	**(0.80,0.94)**	0.94	(0.86,1.04)
Diet (ref. high-sodium diet)												
Middle-sodium diet	**0.86**	**(0.79,0.94)**	**0.86 **	**(0.79,0.93)**			**0.86 **	**(0.76,0.98)**	**0.75 **	**(0.68,0.83)**	**0.84 **	**(0.76,0.94)**
Low-sodium diet	**0.70**	**(0.63,0.79)**	**0.70 **	**(0.64,0.77)**			**0.78 **	**(0.67,0.90)**	**0.62 **	**(0.55,0.69)**	**0.71 **	**(0.62,0.80)**
Driving a car (ref. yes)												
No	**0.70**	**(0.63,0.78)**	**0.73 **	**(0.64,0.83)**	**0.74 **	**(0.66,0.83)**			**1.26 **	**(1.16,1.37)**	0.93	(0.79,1.10)
Subjective health status (ref. poor)												
Good	**0.62**	**(0.51,0.74)**			**1.20 **	**(1.05,1.36)**	**0.65 **	**(0.53,0.79)**	**0.86 **	**(0.77,0.96)**	0.95	(0.86,1.05)
Stress perception (ref. no)												
Yes	**1.19**	**(1.09,1.29)**	1.05	(0.97,1.13)			**1.30 **	**(1.16,1.45)**	**1.17**	**(1.07,1.27)**	1.00	(0.90,1.11)
Depressive symptoms (ref. no)												
Yes	1.03	(0.84,1.26)					1.19	(0.99,1.42)	0.97	(0.85,1.11)		
Number of chronic illnesses (ref. 0)												
1	**2.49**	**(2.18,2.86)**	**1.74 **	**(1.60,1.88)**	**1.60 **	**(1.40,1.83)**	**2.04 **	**(1.62,2.55)**	**1.92 **	**(1.75,2.10)**	**1.39 **	**(1.21,1.60)**
≥2	**4.97**	**(3.57,7.00)**	**2.84 **	**(2.56,3.15)**	**2.64 **	**(2.30,3.02)**	**3.95 **	**(2.19,7.04)**	**3.49 **	**(3.11,3.92)**	**2.47 **	**(2.17,2.81)**
Distance to public physical activity facilities (ref. T1 (nearest))												
T2	1.10	(1.00,1.20)	**1.13 **	**(1.03,1.23)**			1.04	(0.89,1.21)	1.00	(0.90,1.12)		
T3 (farthest)	1.01	(0.90,1.13)	1.12	(0.99,1.26)			0.92	(0.73,1.14)	1.07	(0.90,1.26)		
Distance to public parks (ref. T1 (nearest))												
T2							1.10	(0.95,1.27)	1.07	(0.95,1.19)		
T3 (farthest)							**1.33**	**(1.05,1.69)**	1.14	(0.94,1.38)		
Distance to public transit (ref. T1 (nearest))												
T2					1.15	(1.00,1.31)	1.07	(0.91,1.26)	0.94	(0.84,1.07)		
T3 (farthest)					1.06	(0.92,1.22)	0.98	(0.79,1.21)	1.01	(0.85,1.21)		
Population density (ref. T1 (lowest))												
T2			**0.88 **	**(0.79,0.98)**			1.18	(0.95,1.46)	0.89	(0.76,1.04)		
T3 (highest)			0.97	(0.86,1.10)			0.93	(0.71,1.22)	0.86	(0.70,1.05)		
Intersection density (ref. T1 (lowest))												
T2									1.10	(0.97,1.24)		
T3 (highest)									1.06	(0.92,1.21)		
Land use mix (ref. T1 (lowest))												
T2	1.01	(0.92,1.11)					1.04	(0.91,1.20)	1.03	(0.93,1.14)		
T3 (highest)	1.09	(0.99,1.21)					0.97	(0.83,1.14)	0.97	(0.86,1.09)		
Deviance information criterion	15,227.78		19,605.13		8613.18		9904.93		17,077.72		11,290.27	
Spatial fraction within community	0.467		0.495		0.475		0.991		0.038		0.565	

OR, odds ratio; 95% CI, 95% confidence interval; MVPA, moderate and vigorous physical activity; ref., reference category; T, tertile.
